# Assessing the Impact of Marine Tourism and Protection on Cultural Ecosystem Services Using Integrated Approach: A Case Study of Gili Matra Islands

**DOI:** 10.3390/ijerph191912078

**Published:** 2022-09-24

**Authors:** Urai Ridho A. M. F. Banarsyadhimi, Paul Dargusch, Fery Kurniawan

**Affiliations:** 1 School of Earth and Environmental Sciences, University of Queensland, Brisbane, QLD 4072, Australia; 2 Center for Coastal and Marine Resources Management of Pontianak, Directorate General of Marine Spatial Management, Ministry of Marine Affairs and Fisheries Republic of Indonesia, Pontianak 78114, West Kalimantan, Indonesia; 3 Department of Aquatic Resources Management, Faculty of Fisheries and Marine Sciences, IPB University (Bogor Agricultural University), Bogor 16680, West Java, Indonesia; 4 Center for Coastal and Marine Resources Studies, IPB University (Bogor Agricultural University), Bogor 16680, West Java, Indonesia

**Keywords:** cultural ecosystem services, hedonic, eudaemonic, marine tourism, marine protection, marine protected area, small islands, coastal, Indonesia

## Abstract

Cultural ecosystem services (CES) are intangible benefits people obtain from an ecosystem through physical and cognitive interactions. Understanding CES provides vital insights into how activities impacting ecosystem services also impact people. Gili Matra Islands, a set of three small tropical islands located in West Nusa Tenggara Province, Indonesia, are an increasingly busy marine tourism destination and a marine protected area. By integrating a hedonic monetary value model with a eudaemonic non-monetary value model, this study examines the impacts of tourism and marine protected area management on cultural ecosystem services in the Gili Matra Islands. Results showed that the distance had significantly influenced property prices to coastlines, beach spots and coastlines with sunset views. In addition, the property prices of each individual island showed significant correlations with particular marine tourism and protection features. Less restricted marine protected zones and coastlines were the most significantly influencing variables to the strong eudaemonic well-being dimensions expressed by residents. The Spiritual dimension produced the highest score and was most significantly affected by several features. This study utilised higher accuracy of properties and residents’ location, enabling more accurate assessments of interaction between CES and the features. This study also discusses how these novel insights in the small island’s CES case can inform vulnerability assessments, reviews of recreation taxes, and spatial planning for marine protected areas and help optimise beach nourishments.

## 1. Introduction

Ecosystem services are defined as “benefits people obtain from ecosystems” [1]. Marine and coastal environments provide a wide range of ecosystem services, including fisheries, food, tourism, spiritual opportunity, genetic material, coastal protection, biodiversity, fish habitat, climate regulation, and so forth [2,3]. Marine and coastal environments also provide cultural ecosystem services (CES) that are largely unexplored. CES arises through physical and cognitive interactions between the natural environment and people and includes intangible benefits people obtain, such as spiritual, aesthetic, and recreational experiences [4,5]. CES is more challenging to assess than other ecosystem services, which might make CES the least explored [4]. However, CES research is emerging, with CES becoming more recognised for providing benefits to people [6], particularly when more complex conflicts occur when CES is overlooked [4,7]. In marine and coastal environments, research has focused mainly on economic valuations of nature-based tourism [6], while research into CES, like spiritual interaction and others, is limited [8]. When evaluating human well-being in response to different ecosystems, CES assessment should involve diverse measures [9]. Notably, coastal areas, particularly tropical islands, are more likely to have varied and cross-boundary CES due to intensive interactions between tourism activities, the natural environment, indigenous and local communities, and other factors [10]. Appropriately comprehending the CES will enable ecosystem services sustainable management whilst also improving people’s well-being [11].

Due to its multi-dimensionality, research into CES requires multiple assessments facilitated by integrated methods. Integrating hedonic and eudaemonic approaches is effective for assessing both monetary and non-monetary values of CES, for instance, a study on Scotland’s west coast investigated the interrelation effect of marine aquaculture and marine protection to CES values reflected in properties prices and resident’s eudaemonic well-being [4]. The hedonic approach defines well-being in terms of happiness, pleasure attainment, or simply, life satisfaction [12]. In this study, the hedonic pricing model is used to estimate the influence of marine tourism and marine protected areas (MPAs) on marine and coastal environment CES through monetary value in the form of property prices. The property market is an excellent economic indicator for ecosystem services for its direct relation with the market [13]. Subsequently, a eudaemonic approach is adopted in this study to define life quality through people’s best potential, personality, and fulfillment [14], and to conceptualise relational values between nature and people [15]. CES benefits in marine areas are operationalised as aspects of human well-being as a result of interaction between marine settings and cultural activities [16]. Another dimension of CES can also be seen in people’s positive mindset on their social media activity during their visitation to MPAs site, which can be measured by social web photo repository sampling [17].

This study aims to understand the influence of marine tourism activities and marine protection on CES, particularly for small tourism islands, and assess the monetary and non-monetary value of these CES. The uncontrolled anthropogenic activities of marine tourism may threaten not only the natural environment but also the CES [4]. This CES’s degradation can be consequently reflected through their monetary and non-monetary value. Conversely, marine protection effort such as MPAs aims to conserve or even improve environmental quality. MPAs can greatly influence CES’s provision such as people’s behavior and social value both spatially and temporally [18,19]. People can even benefit from MPA as simple as having a positive mindset on their social media when situated within the MPA [17]. These two opposite activities are very likely to happen on tourism islands, causing the CES’s state to become more complicated to assess [4,20]. To achieve this, hedonic and eudaemonic well-being approaches were adopted in this study. Property prices for the hedonic model and subjective well-being responses for the eudaemonic model were obtained through questionnaires and online markets, while remote sensing data supplied the features of interests, such as coordinate position and digital elevation model (DEM). Remote sensing (RS) and geographic information systems (GIS) are capable of acquiring spatial data about environmental monitoring, land–water surveys, changes, and ecosystem services, both spatially and temporarily [21,22]. Given the limited research into CES associated with marine and small island areas, this study will help support the policy-making and conflict resolution for sustainable tourism and environmental protection and other management systems in this region, so that local communities, tourism industries, and natural ecosystems can thrive together in the future. To achieve the research aims, the study was conducted according to the following objectives:

1. Use a hedonic approach to assess the monetary value of CES influenced by marine tourism and marine protection in the Gili Matra Islands.
2. Use a eudaemonic approach to assess well-being values associated with CES in marine and coastal environments in Gili Matra Islands.
3. Investigate the interrelations between CES-associated well-being value to marine tourism and marine protection in Gili Matra Islands.

## 2. Materials and Methods

### 2.1. Study Area: Gili Matra Islands

**Figure 1:**
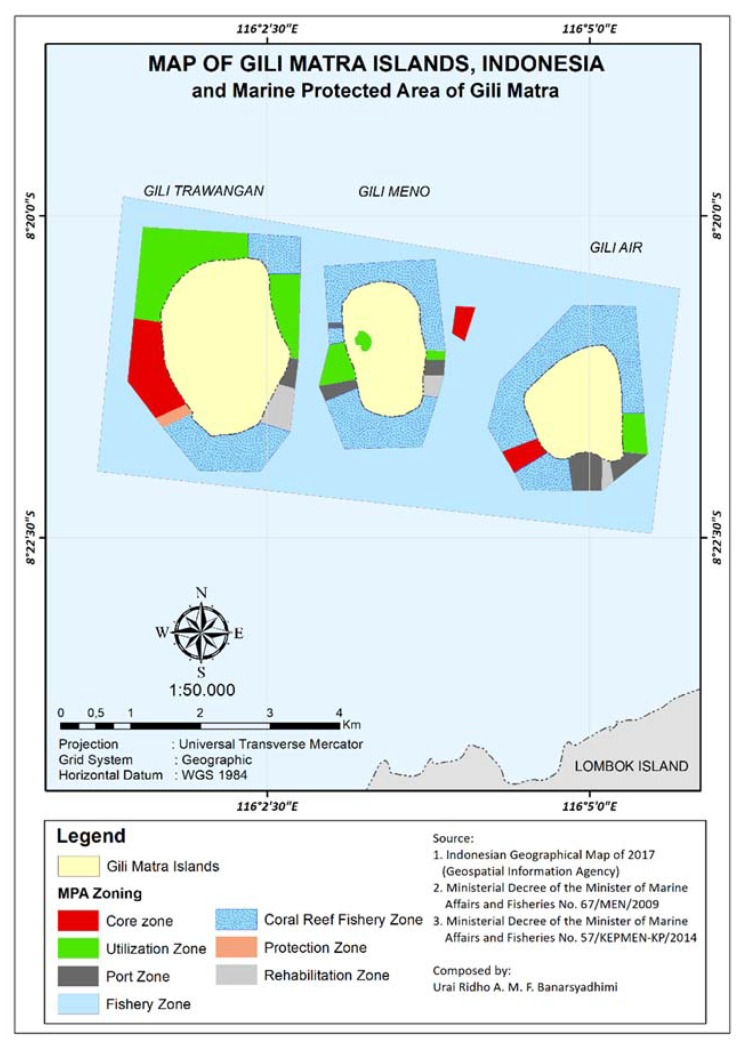
Adapted Map of Marine Protected Area of Gili Matra Islands [25].

Gili Matra Islands (Figure 1), West Nusa Tenggara, Indonesia, were chosen as the case study to represent a marine protected area and marine tourism destination [23]. The name “Gili Matra” is an abbreviation of Gili Meno, Gili Trawangan, and Gili Air—three islands that provide a home for many marine tourism sites, such as coral reefs, scenic beaches, etc. To help sustain the marine and coastal resources, the government of the Republic of Indonesia designated a marine protected area (MPA) for the islands—a geographical space defined and managed through legal or other effective means to achieve the long-term conservation of nature and associated ecosystem services and cultural values [24]. Its primary function as a Marine Tourism Park is to promote sustainable nature-based tourism while conserving the natural environment so the society can benefit from the area [25,26,27].

**Figure 2:**
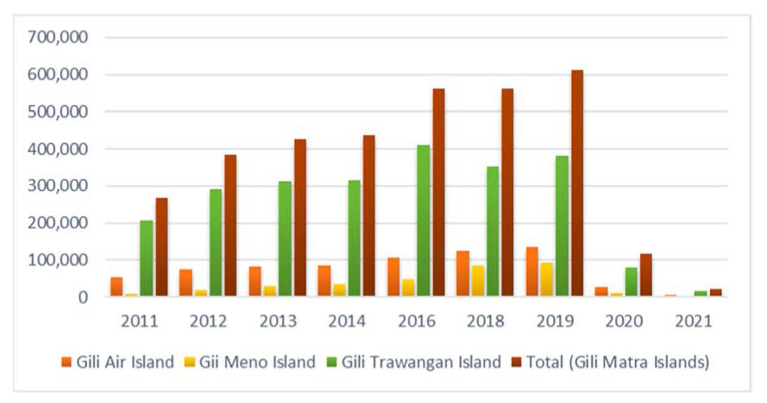
Tourists increased in Gili Matra Islands. Adapted with permission from Ref [30,31]. 2021, Dept. of Tourism.

**Figure 3:**
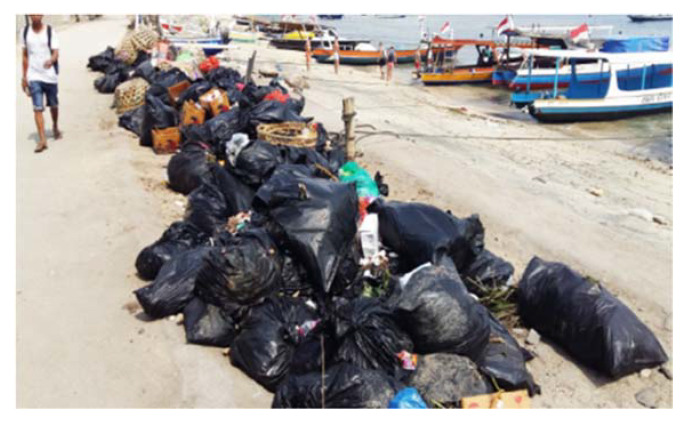
Mounds of waste by settlement and tourism accommodation at Gili Air’s coast, one of the Gili Matra Islands [29].

Since tourism emerged in 1986 [28], Gili Matra Islands have experienced increasing tourism activities, predominantly landscape changes for residential and tourism accommodation [23,29,30]. Gili Matra Islands have developed unique and diverse CES like other coastal tropical islands that have undergone increasing tourism activities [10]. The number of tourists and residents reportedly increased, as shown in Figure 2 [23,30,31], except during the COVID-19 pandemic. Unfortunately, these increases imposed direct (e.g., physical damage) and indirect stressors (e.g., pollution waste, as shown in Figure 3) that degraded ecosystem and seawater quality [23,29,30,32,33,34]. This problem becomes even more critical given the increasing anthropogenic value of tourism interacting with CES. That is, negative impacts may degrade the ecosystem and the CES, such as marine recreational, aesthetic value, spiritual value and other cultural uses [4].

### 2.2. Data

#### 2.2.1. Questionnaire

A questionnaire was prepared to collect information about residential property prices, locations, and well-being values associated with CES. The questionnaire aimed to extract hedonic and eudaemonic primary data from the residents of Gili Matra Island through 4 general questions, 6 hedonic well-being open-questions, and 23 eudaemonic a 7-point Likert scale questions. The survey managed to collect 131 responses for hedonic data and 132 responses for the eudaemonic instrument. The questionnaire can be found in Supplementary Materials Questionnaire S1.

#### 2.2.2. Secondary Data

1. Tourism accommodation property prices and locations from online markets.
2. Marine tourism sites, like a coastline, beach spots, and dive spots, from Google Maps [35], OpenStreetMap [36], and literature.
3. Marine protected area (MPA) zoning [25].
4. Digital elevation model (DEM) [37,38].
5. Public facilities and road network [29,30].

### 2.3. Methodology

**Figure 4:**
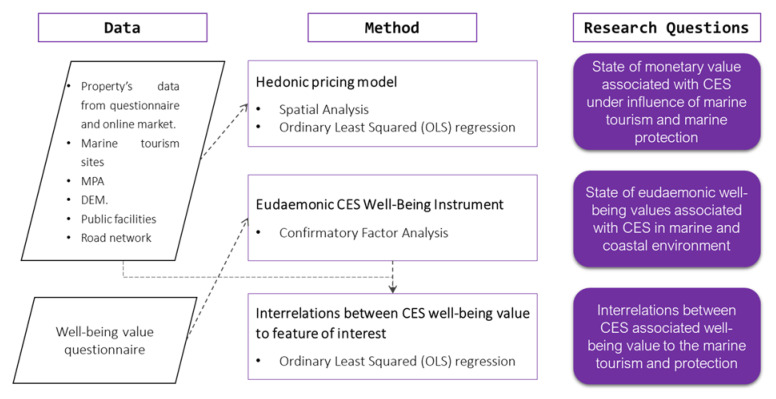
Methodology and data for respective research question.

Statistical and spatial analyses were conducted according to the methods described in Figure 4. Software included SPSS Statistic 26, SPSS AMOS 25, QGIS 3.18.2, and ArcGIS Pro.

#### 2.3.1. Hedonic Approach to Assess the Monetary Value of CES Influenced by Marine Tourism and Marine Protection in the Gili Matra Islands

The hedonic pricing model examined the impact of the features of interest on people’s selections, which were property prices representing the monetary value of CES [4]. The features of interest included marine tourism sites (i.e., coastline, sunset view coastline, beach spots, and dive spots), marine protected area (MPA) core zone (strict zone) and another zone (less strict), public facilities, and road networks [29,30]. To determine the impact of these features of interest, spatial-environmental attributes such as distance to and visibility were considered [4,13]. They were employed through standard multiple regression statistical analysis using SPSS Statistic 26 [39], as described in Equation (1).

P= *α* + *β*_1_
*E*_1_ + *β*_2_
*E*_2_ (1)

where P is property price, representing the CES’s monetary values; *α* is Intercept (constant term); *β* is the slope coefficient; *E* is attributes of the features of interest.

Unlike previous research [4], this study used property price data from both residential and tourism accommodations (e.g., hotels and cottages). Consequently, separate models were employed for different property types and other variations (i.e., set of islands and individual islands) that provided further insights. Property price and location data for the residential properties were obtained through the questionnaires along with well-being questionnaires (with assistance from a GPS device for high accuracy of location). For primary data/residential property prices, a total of 131 observations were collected. Subsequently, tourism accommodation property prices and addresses were compiled from online markets. Keywords included ‘Gili Matra’, ‘Gili Trawangan’, ‘Gili Air’, ‘Gili Meno’, and so forth. Data from 46 tourism accommodation properties were collected. Prior to statistical analysis, the attributes of each feature of interest, i.e., distance and visibility, were derived through spatial analysis using QGIS 3.18.2 (QGIS Association) and ArcGIS Pro (ESRI, Redland, CA, USA), which required location data and Digital Elevation Model (DEM). The DEM was collected from Indonesia Geospatial Portal [37] and the United States Geological Survey database [38]. The inverse distance weighted (IDW) method was then used to interpolate property price data and visualise data distribution.

#### 2.3.2. Eudaemonic Approach to Assess Well-Being Values Associated with CES in Marine and Coastal Environments in Gili Matra Islands

To investigate the value of marine tourism and protection features to marine users, the well-being value questionnaire was based on an instrument previously developed by the UK National Ecosystem Assessment (NEA) [16] and other studies [40,41,42]. The instrument incorporated seven dimensions of well-being influenced by CES, including Place identity, Therapeutic value, Engagement and interaction with nature, Social bonding, Memory/transformative value, Spiritual value, and Challenge and Skill. Each dimension was associated with several indicators investigated by exploratory factor analysis [4,16]. Using the instrument, respondents assigned a value to each indicator on a 7-point Likert scale regarding their agreement with natural places nearby, reflected from 1 (strongly disagree) to 7 (strongly agree). Target respondents were the residents of the Gili Matra Islands, and a total of 132 responses were collected.

Statistical analysis of the collected data was performed through confirmatory factor analysis (CFA) using SPSS Amos 25 and SPSS Statistic 26 (IBM, Armonk, NY, USA). CFA was chosen because well-being indicators and dimensions were based on previous literature rather than through own exploration [4]. This analysis investigated relationships between these indicators and well-being dimensions associated with marine and coastal CES. The model was evaluated through several fitness tests, i.e., Chi-Square statistic and degree of freedom (CMIN/df), comparative fit index (CFI), Tucker–Lewis index (TLI), root mean squared error of approximation (RMSEA), and standardised root means squared residual (SRMR). The indicators are provided in Appendix A, Table A1. The questionnaire template for the eudaemonic and hedonic model is available in Supplementary Materials Questionnaire S1.

#### 2.3.3. Interrelations between CES Associated Well-Being Value to Marine Tourism and Marine Protection in Gili Matra Islands

Correlations between CES-associated well-being scores and features of interest were performed through standard multiple regression modelling using SPSS Statistic 26 [39]. Correlations determined if there were influences from the features of interest on respondent valuation over their CES-related well-being related. The dependent variable was the factor scores for each dimension of CES well-being, and the independent variables were the distance to and visibility of features of interest. Since seven dimensions of CES well-being were adapted [4], the models were run seven times, corresponding to each factor.

## 3. Results

### 3.1. Influence of Marine Tourism and Marine Protection on the Monetary Value Associated with CES

The first model (model HR1) of residential property prices used price data for the three islands as a dependent variable. Table 1 shows the statistical summary. The minimum price of residential properties was found at 75 million Indonesian Rupiah (IDR) per are (1 are equals to 100 square meters (100 m^2^)) (100 m^2^), while the highest price was 1000 million IDR per are. The closest feature was the road with almost no distance (only 0.97 m), while the furthest feature was the core zone (MPA’s strict zone) with 1620.35 m. The roads themselves are always visible to every residential property. An ANOVA/F-test showed that the model reached statistical significance (Sig. = 0.000), and the R^2^ value was considerably high (0.641), implying that the independent variables collectively explained 64.1% of the variance in the dependent variable [39]. Three variables had a significant individual contribution, i.e., distance to the coastline, distance to beach spots, and visibility of the coastline with a sunset view. An increase of one meter in the distance to the coastline decreased the price by 0.697 IDR per are (100 m^2^). Similarly, an increase of one meter to a beach spot lowered the price by 0.128 million IDR per are. Meanwhile, visibility of at least one coastline facing the sunset increased price by 143.95 million IDR per are. The regression equation and *t*-test for Model HR1 are shown in Supplementary Materials (Equation (S1) and Table S1).

**Figure 5:**
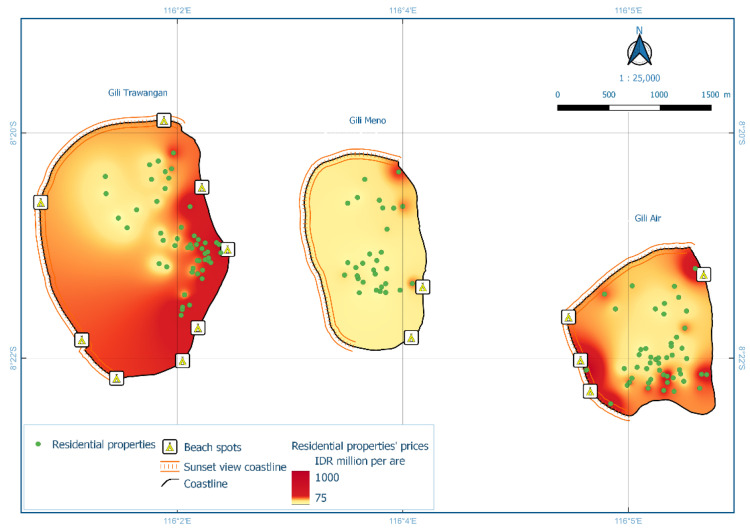
Interpolation of residential property prices for Gili Matra Islands (Model HR1).

Figure 5 reveals that high prices were generally located near the coastline. Spatial interpolation also indicated that lower values or price within the first quartile (<25%) resides in the middle of the island, far from the beach spot, coastline, or other marine tourism sites, as shown in Table 2. On the other hand, higher property prices, i.e., prices in the third (50–75%) and fourth (75–100%) quartile, were mostly located near the coastline. Each island also displayed a unique distribution pattern for properties with higher prices.

**Figure 6:**
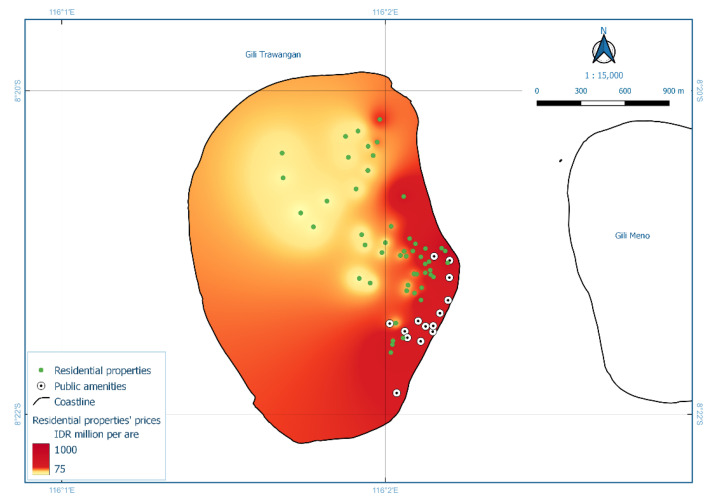
Interpolation of residential property prices for Gili Trawangan Island (Model HR2).

The second model, Model HR2, used residential property prices for Gili Trawangan Island. ANOVA/F-test showed that the model was statistically significant (Sig. = 0.000) [39], with the R^2^ value was 74.4%. Two independent variables had a significant individual contribution, i.e., distance to coastline and distance to facilities. An increase of one meter in the distance to the coastline decreased property prices by 1.011 million IDR per area. Furthermore, a one-meter increase in the distance to public facilities decreased residential property prices by 0.464 million IDR per are. Spatial distribution is presented in Figure 6. The regression equation and *t*-test for Model HR2 are provided in Supplementary Materials (Equation (S2) and Table S2).

**Figure 7:**
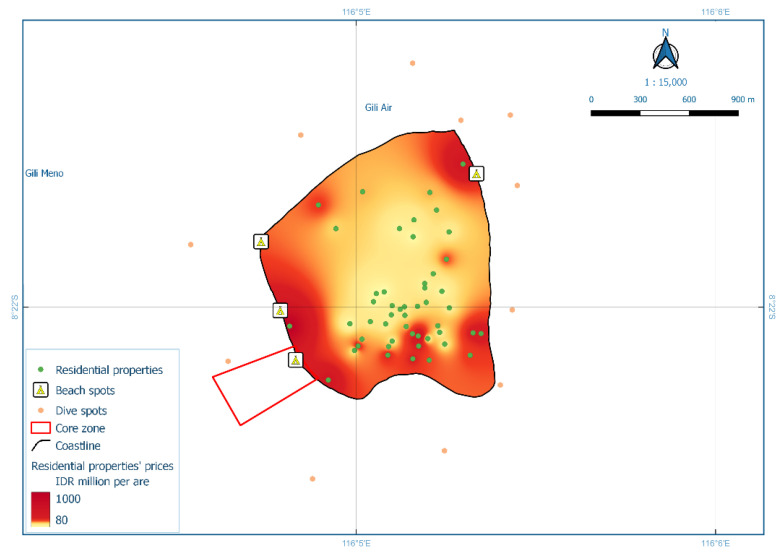
Interpolation of residential property prices for Gili Air Island (Model HR3).

**Figure 8:**
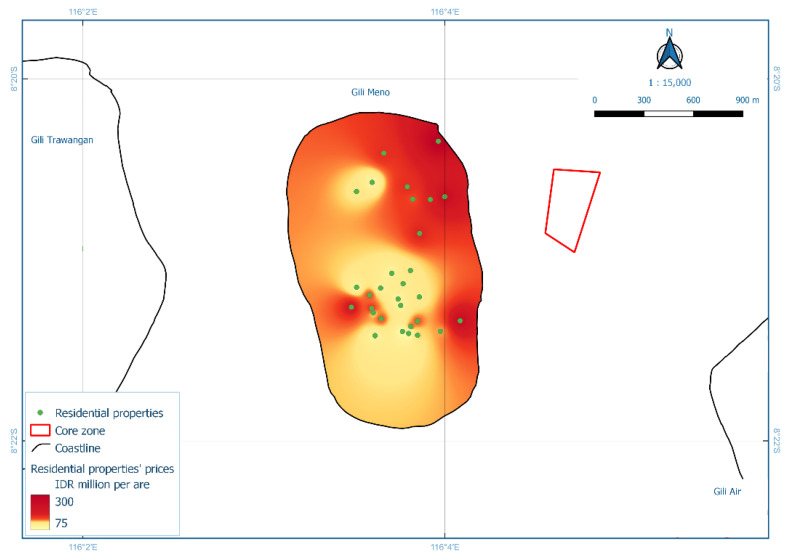
Interpolation of residential property prices for Gili Meno Island (Model HR4).

The third model, Model HR3, analysed residential property prices for Gili Air Island. An ANOVA/F-test showed that the model was statistically significant, and the revealed R^2^ value was 0.729. Four independent variables had significant individual contributions (*t*-test with Sig. < 0.05, [39]), i.e., distance to beach spots, distance to dive spots, distance to core zone, and distance to the road. An increase of one meter in the distance to beach spots lowered residential property prices by 0.467 million IDR per are, as did the distance to dive spots decreased prices by 1.003 million IDR per are for every one-meter increase. Increased distance to the core zone of MPA also lowered prices by 0.454 million IDR per are for every one-meter increase. The most substantial impact on property prices was observed in the distance to the road, which reduced prices by 2.004 million IDR per are for every one-meter increase. The spatial distribution of these findings is presented in Figure 7. The fourth model, Model HR4, analysed residential property prices for Gili Meno Island. An ANOVA/F-test showed that the model was statistically significant, and the R^2^ value was 0.876. Two independent variables had significant individual contributions. Increased distance to the coastline decreased prices by 0.271 million IDR per i for every one-meter increase. Similarly, increased distance to the MPA core zone decreased prices by 0.140 million IDR per are for every one-meter increase. The spatial distribution of these findings is presented in Figure 8. The regression equation and full *t*-test for Model HR3 are available in Supplementary Materials in Equation (S3) and Table S3, whereas for Model HR4 are in Equation (S4) and Table S4.

**Figure 9:**
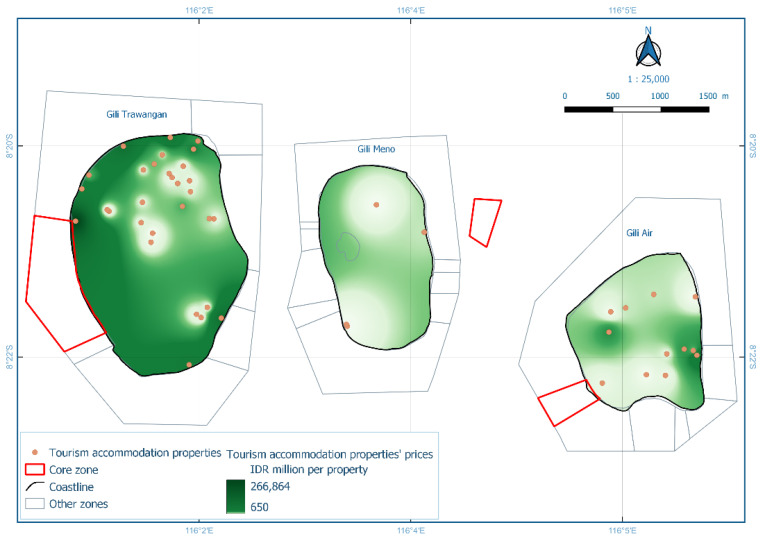
Interpolation of tourism accommodation property prices (Model HT1).

The final model in the hedonic analyses, Model HT1, analysed property prices of tourism accommodation derived from secondary data. Model HT1 included 46 properties across all three islands. Unlike the residential property, tourism accommodation property prices were expressed per whole property rather than per are (100 m^2^). Table 3 shows a statistical summary for Model HT1. An ANOVA/F-test showed that the model reached statistical significance (Sig. = 0.000), revealing that all independent variables collectively significantly influenced the prices. The R^2^ value of Model HT1 was 0.826. However, only one independent variable, bedroom number, had a significant individual contribution (*t*-test Sig. < 0.05) to predicting accommodation property prices. Subsequently, due to marine tourism and protection features not significantly contributing to property prices in Model HT1, the regression model was repeated with gradual eliminations of the independent variables. Five omitted variables were selected in order from least significant to most significant; visibility of the road, distance to beach spots, visibility of beach spots, distance to dive spots, and visibility of dive spots. The new regression produced four significant independent variables. Distance to the coastline facing the sunset decreased tourism accommodation property prices by 43.686 million IDR for every one-meter increase. The visibility of at least one core MPA zone increased prices by 29,446.45 million IDR, while the visibility of at least one other MPA zone increased prices by 30,042.83 million IDR. Lastly, the addition of one-bedroom increased prices by 2445.998 million IDR. Figure 9 shows a spatial interpolation of Model HT1. The regression equation and full *t*-test for Model HT1 are provided in Supplementary Materials (Equation (S5) and Table S5).

### 3.2. Eudaemonic Well-Being Values Associated with CES in Marine and Coastal Environments

Kaiser-Meyer-Olkin (KMO) and Bartlett’s test results showed that the KMO Measure of Sampling Adequacy value was 0.864 (threshold of >0.6) and Bartlett’s Test of Sphericity Sig. value was 0.000 (threshold of <0.05), which meant that the factor analysis model (Model E1) was appropriate and statistically significant [39]. Next, the model fit measure and validity tool test by the SPSS Amos plugin [43,44] showed that the model could be improved by removing several indicators, including Eng3, Eng5, Plid 8, and Soc17 (see Appendix A Table A1). Following the removal of these indicators in Model E1, communalities values were robust (>0.3), ranged from 0.446 to 0.852 (see Supplementary Materials Table S6), showed that these indicators fitted well with other indicators and significantly explained total variances in Model E1 [39]. Several goodness of fit tests were used and are presented in Supplementary Materials Table S7. Three tests showed good results in standardised root means squared residual (SRMR), Chi-square (CMIN), and Degree of Freedom (df). The SRMR presented a value of 0.074, which was less than 0.08, denoting that the model was a relatively good fit between the observed data and the hypothesised model [45]. The CMIN/df value was 2.155, less than 3, indicating an acceptable [46] and reasonable fit (<5) [47] between the sample data and the model.

**Figure 10:**
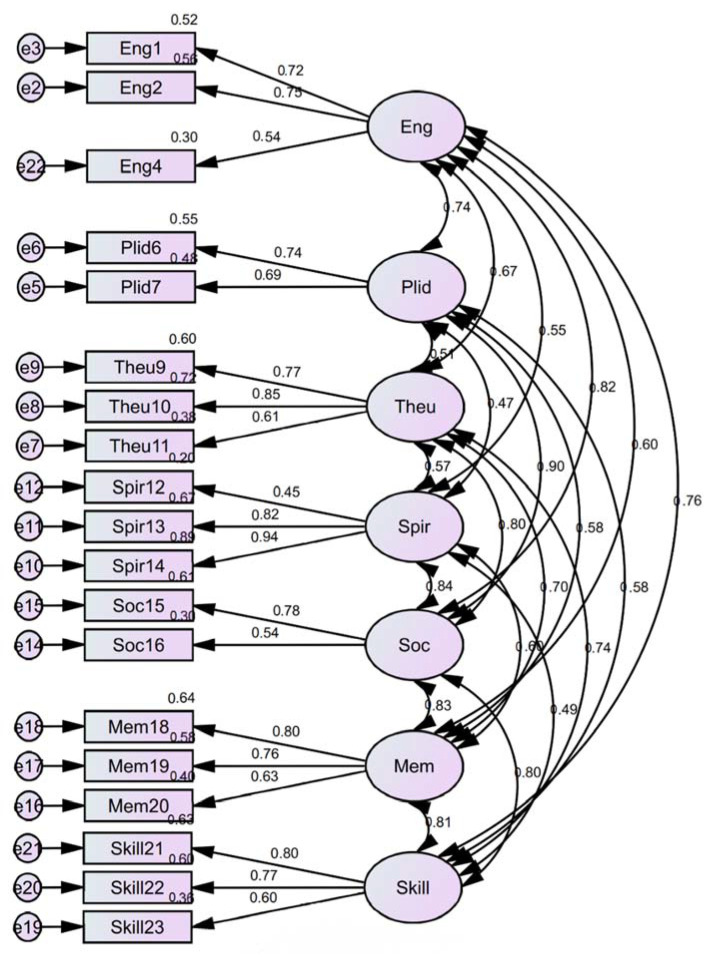
Structure of Model E1 using CFA analysis.

**Figure 11:**
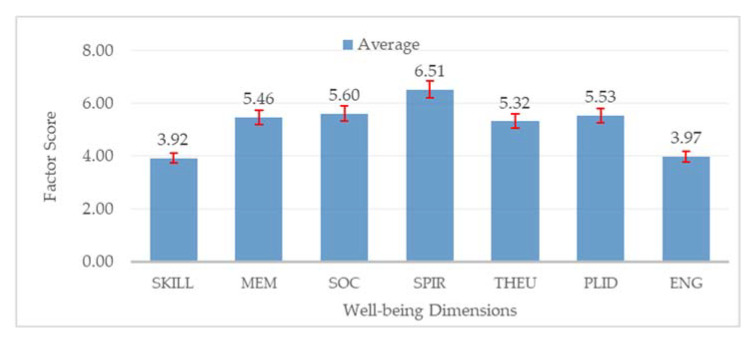
Overall factor scores average and standard deviation for Gili Matra Islands. SKILL: Skill and challenge dimension; MEM: Memory/Transformative value; SOC: Social bonds; SPIR: Spiritual value; THEU: Therapeutic value; PLID: Place identity; ENG: Engagement and interaction with nature.

Indicator loadings values were mostly above 0.6, implying a valid indicator and its solid association with the respective well-being factor [4], as shown by the values on arrows from each dimension to corresponding indicators in the structure model in Figure 10. For instance, the loading factor from the Spiritual dimension to indicator “Spir14” was the highest, indicating that this indicator most represents the well-being dimension expressed by the local residents. The CES well-being factor scores for each response were then calculated using the factor score weight produced by the CFA analysis (see Supplementary Materials Table S8). For instance, the Skill factor score for response number One was calculated by the equation in Supplementary Materials Equation (S6). After calculating each factor and response, the average and standard deviation of the factor score were calculated for overall responses, as shown in Figure 11.

**Figure 12:**
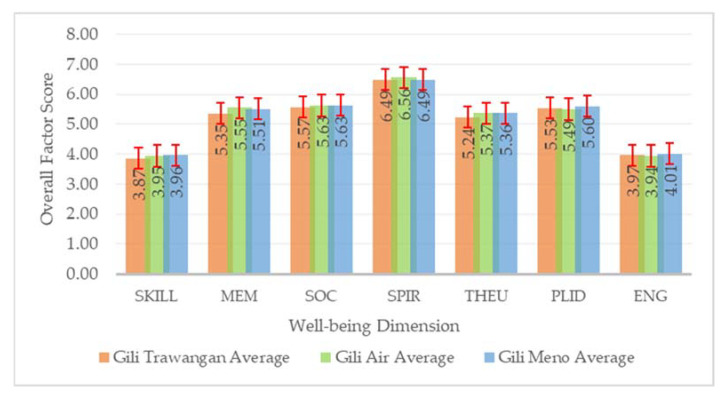
Overall factor scores averages and standard deviation for each island.

The average CES well-being dimension scores were high. The lowest factor score was 3.92, still above the midpoint (3.5). These results suggested that the residents of Gili Matra Island experienced positive impacts from CES associated with marine and coastal environments. Among all dimensions, Spiritual value received the highest score with a value of 6.51, inferring that the residents benefited most from CES through their spiritual aspect. The next highest scores were Social bond, Place identity, Memory value, Therapeutic value, Engagement and interaction with nature, and Skill and achievement. Notably, each island also showed the same patterns as shown in Figure 12.

### 3.3. Interrelations between CES Associated Well-Being and Marine Tourism and Protection

Subsequent analyses investigated the correlation between well-being values and previously utilised marine tourism and protection features in the hedonic monetary value analyses. The regression ran seven times, one for each of the CES well-being dimensions used. The first model of this analysis, Model R1, used the first dimension, Skill and challenge value, as the dependent variable from 132 responses. The result of the ANOVA/F-test suggested that the model was not statistically significant in collectively affecting the factor score of Skill values. The independent variables also could only explain 16.4% (R^2^ was 0.164) of the variances in the factor score. However, two independent variables did have significant individual contributions to Skill values—distance to coastline and distance to other zones. One-meter increases in the distance to the coastline increased the Skill values score by 0.002, while one-meter increases in the distance to other MPA zones decreased the Skill values score by 0.002.

Correlations between all well-being dimensions and marine tourism and protection features were similar. Table 4 summarises the impact of each independent variable on each well-being factor/dimension, along with their R^2^ values. Even though the models did not collectively explain the influences on the factor scores, there were consistent significant individual contributions from several independent variables to the respective well-being dimension. That is, there were consistent significant individual contributions by distance to other zones and distance to the coastline. As the distance to other zones (i.e., the less strict zones) increased, the score for every CES well-being factor decreased. Conversely, the further distance to the coastline, the higher the factor score. For the core zone (no-take zone), the influence only emerged for Spiritual value, where the score dropped as the distance increased. Meanwhile, minimal impacts (i.e., a coefficient value of zero or almost zero) were shown by several independent variables, including distance to the coastline facing the sunset, distance to the dive spot, distance to the core zone, and distance to public amenities.

## 4. Discussion

### 4.1. Influence of Marine Tourism and Marine Protection on the Monetary Value Associated with CES

The first model, Model HR1, indicated that the independent variables used were mostly comprehensive and covered various marine tourism and protection features. Additionally, the infrastructures that support tourism activities in the area include a road network, road accessibility, and amenities [30,48,49,50,51]. Housing prices can indeed represent the benefits gained from close proximity to coastlines, like beach aesthetics, therapeutic, and others [4,52]. That is, house owners are willing to pay (WTP) a higher price to enjoy the CES provided [52,53,54]. This observation is supported by Table 2, where the highest prices were found to be located adjacent to the coastline. The strong impact of beach spots and coastline on residential property prices should also be a warning about depleting the coastal environment by mass utilisation. Following a WTP appraisal, mechanisms like zoning plans or recreation fees could be improved to hinder the depletion [53]. Visibility of the coastline facing the sunset also significantly influenced residential property prices. The sunset view might enhance property prices [55] as buyers are willing to pay more for the premium views [56,57]. However, other visibility features did not significantly influence. This may be due to the flat terrain of Gili Matra Islands [58,59] reducing visibility.

Subsequently, the model for each island displayed particular characteristics where independent variables (i.e., features) significantly affected residential property prices. In Model HR2, Gili Trawangan, amenities were distributed mainly on the island’s east coast (Figure 7), including the tourism commercial centre, school, etc. [30]. This finding supported the hypothesis and previous research that considered amenities to support coastal tourism infrastructure [48,49,51]. These findings provide evidence of ‘hot spots’ for economic activities and settlement. For instance, based on the high property prices dominating the eastern coast of Gili Trawangan, a cost–benefit analysis may recommend beach nourishment to prevent erosion in premium locations [53,60].

Model HR3 suggested that there were four significant variables in Gili Air Island: distance to dive spots, distance to road, distance to beach spots, and distance to the MPA core zone. Eleven dive points on Gili Air were spread evenly around the island, as shown in Figure 8 [29]. This is likely due to local awareness of the recreational activities, biodiversity, and other values that dive spots offer. The distribution of high residential property prices and their correlation with nearby dive spots (coral reefs) could be used as a novel input for updating vulnerability assessments. The latest vulnerability assessment on the island used parameters such as coastline changes, live coral changes, coral reef changes, and developed areas [29]. An accessible road network is a major determinant of nearby property prices [61,62,63]. Roads are considered crucial infrastructure for supporting tourism development [49,51], particularly in Gili Matra Islands, where gas-fueled vehicles are prohibited, making accessible roads essential for pedestrians and cyclists.

Model HR4 showed that distance to the coastline and core zone significantly influenced property prices in Gili Meno Island (Figure 9). The core zone is a “no-take” zone in MPA with strict protection and very minimum human activity [64,65,66], thus, is more likely to have more biodiversity [66]. Although these benefits may not be visible and could not be directly enjoyed, the residents in Gili Matra were most likely aware of these protected areas and related conservation activities [29,67], which may convince them to pay more for properties closer to marine conservation sites [4,68]. The impact of core zones observed in this study may prompt updating of MPA zoning.

According to Model HT1, it is reasonable that hotel or tourism accommodation properties that provide tourists with water views would receive higher prices [69,70]. Figure 10 shows how higher prices were generally situated closer to the coastline in Gili Trawangan. However, this pattern was not obvious in the other two islands, suggesting other variables were dominant. Lastly, the influence of bedroom number on property price was strongly evident as it is one of the primary features that any tourist seeking accommodation considers. The results from hedonic models used in this study showcased various patterns across different islands and property types. These multiple models managed to reveal the impact of distinct marine tourism and protection features on people’s valuation of their properties. Each island displayed its own price pattern and what unique features influenced this pattern, which subsequently became highly crucial for island and water management.

### 4.2. Eudaemonic Well-Being Values Associated with CES in Marine and Coastal Environment

For all six CES well-being dimensions, the high score displayed by the results indicated that residents of Gili Matra Island have been experiencing positive impacts from CES. The residents of Gili Matra Islands experienced the most benefit from Spiritual value (Figure 11). This result varied from previous research by Spanou et al. [4] where the highest score was Therapeutic value. The respondents in Spanou’s research were local residents in Argyll and Bute, a region on Scotland’s scenic west coast [4,71]. The picturesque coastal area most likely contributed to the therapeutic value experienced by the locals as they can benefit every day. Meanwhile, for locals in Gili Matra, it appeared that the scenery of the islands impacted their well-being mainly on a spiritual level. Spirituality is strong among the people of Gili Matra Islands and Lombok Island, who are well known for practising their religion and living harmoniously [72,73,74]. A coastal environment may enhance spirituality by promoting outdoor activities that enable people to enjoy a scenic natural environment, away from the hustle of cities [75].

The social bond is one of the most well-preserved values in most Indonesian communities. Furthermore, Gili Matra Islands cover only 665 hectares [76], possibly promoting closer and more frequent social interactions between residents. The residents were also found to have community social capital and resilience through iterative and communal activities [77]. This solid social network grew from groups in the islands and even affected governance institutions that consider social and ecological states [78]. Subsequently, the locals were found to highly value the economic aspect of the coastal environment and its surrounding MPA [67]. In addition, a number of marine tourism events have been held, providing avenues for locals and tourists to exchange with each other. These activities have created memories, inspirations, and experiences, both for locals and the tourists in Gili Matra Islands [79].

Despite many respondents showing strong connections with the surrounding natural environment, some expressed uncertainty in taking care of the environment and having their skills improved. These results differed to previous findings [67], suggesting that locals in Gili Matra Islands believed that they value the learning aspect the most. This may be due to different methods. Nevertheless, the eudaemonic models in this study disclosed the residents’ well-being as the result of interaction between CES and surrounding tourism and management features. The discrete pattern between models or dimensions suggested that the eudaemonic models revealed the favoured CES impact on residents’ well-being. In contrast, the similar pattern between islands indicated that the overall results are valid and can be used for the case of small tourism islands. To better understand well-being values in Gili Matra Islands, additional dimensions should be investigated using exploratory factor analysis. Lastly, revisiting the same approach in future research with a larger sample size could also help investigate data consistency and temporal patterns.

### 4.3. Interrelations between CES Associated with Well-Being and Marine Tourism and Protection

The seven regression models for CES-associated well-being dimensions showed significant correlations with two features of interest (Table 4). The other zones in MPA (less strict zone) allow for a wide range of regulated activities, including tourism and education [64]. Besides promoting conservation, a less strict zone also permits more activities and easier access, enhancing place identity and cultural heritage felt by the users [80]. MPAs were able to provide three roles of CES: a place to encourage well-being, a place of spirituality, and a place of preservation and freedom [75,81], or as simple as being able to inspire a positive mindset on social media activity [17]. Based on these findings, managing and shifting the current MPA core zones seaward and increasing the other zones around the island could elevate the CES well-being dimensions of residents. Nevertheless, due to the intangible nature of CES, managers of MPAs may only be able to control the spiritual value of MPAs indirectly [75].

Distance to the coastline showed a negative effect on all well-being dimensions. In Gili Matra Islands, the coastlines were surrounded by a range of MPA zones. This means that those coastlines are regulated, restricting human activities, including core zones (strict, no-take zone) and other zones (less strict zones), which consist of fisheries zones, rehabilitation zones, port zones, protection zones, and utilisation zones [23,25]. The coastline next to or close to the core zone has the most restrictions, as the core zone only allows very limited activities [64,65]. This restricted area might lead to a decrease in the well-being factors observed by people living next to this segment of the coastline. A high proportion (25.05%) of the coastline is adjacent to the core zone, which could also add to the effect on well-being. However, this was not the case for Spiritual well-being and the core zone. Higher proximity to the core zone significantly increased Spiritual well-being. By restricting human activities and preserving natural ecosystems in the core zone, residents may have felt more spiritual well-being through the cleaner water resource [82], quiet situations, open spaces, and natural sounds that the core zone provides [83]. Distance to beach spots and the coastline visibility also impacted spiritual well-being. The beach environments are believed to improve spirituality through outdoor experiences, scenic views, sounds, and the smell of the sea [75].

In Gili Matra Islands, the amenities included tourism commercial centres, schools, and others [30]. These artificial sites presumably detracted from natural ecosystems reducing the spirituality and place identity. Furthermore, artificial structures were considered to lessen well-being and were avoided by people, even though these structures could trigger future development [84]. The urban-like growth across the islands may also impact mental health [85]. Conversely, public amenities provide a space for residents to gather and socialise. This may have been reflected in the finding that visibility of any amenity to the resident could increase Social value. Overall, these results suggested that several features of marine tourism and protection significantly influenced some dimensions of CES well-being felt by the residents. These features, such as coral/dive spots, MPA’s core zones and other features, are unique to Gili Matra Island, which can contribute to constructing local people’s well-being and help establish societal goals and engagement [86]. Some other features and CES well-being dimensions did not show a significant relationship, providing an opportunity for future studies to improve the factor analysis model for assessing CES well-being in small islands.

## 5. Conclusions

This research explored the influence of marine tourism activities and marine protection on CES in small islands with increasing tourism growth by using the Gili Matra Islands as a case study. A hedonic model investigated the CES monetary state reflected in residential property prices. Across the three islands of Gili Matra, higher housing prices were found closer to the coastline, beach spots, and coastlines with a sunset view. When explored separately, each island demonstrated a particular characteristic. The multiple models showing multiple patterns across different islands suggested that although those islands are in the same location, each island’s property prices were influenced by different marine tourism and protection features. These models revealed unique influence and thus specific management action to each island. To further inform the complex influence of any existing features on the island, future research can utilise other types of anthropogenic activities on the island, such as increasing fisheries activities.

The non-monetary state of CES explored in the eudaemonic subjective well-being suggested that residents of Gili Matra Islands experienced significant CES benefits from the island environment. These benefits contributed to several well-being dimensions, primarily to Spiritual value followed by Social bonds and Place identity. All well-being dimensions experienced by the residents showed significant relationships with several features of marine tourism and conservation. The zones in MPA that applied fewer restrictions (i.e., other zones), enabling water activities for residents, showed significant interrelations with all well-being dimensions explored.

### 5.1. Limitations and Future Research

The hedonic CES monetary model’s temporal patterns and changes over time are yet to be explored. Another limitation was the small number of samples collected for tourism accommodation properties. Future research could follow up on the findings, such as the distribution of property prices and dive spots, incorporating them into vulnerability assessment. Future research could also utilise other anthropogenic activities that may influence the price of CES-related properties.

Future research should also explore additional factors relevant to small islands by using exploratory factor analysis in the CES eudaemonic well-being model. More samples and specified categories of respondents may improve outcomes, such as those who directly interact with the features. Lastly, adapting the same methods for future research may help assess temporal patterns and consistency in findings.

### 5.2. Implications for Management and Policy

This study provided several valuable and novel findings that could help inform management decisions. These impacts could be incorporated into the spatial planning and MPA zoning management of the Gili Matra Islands. For example, the correlations between high property price distribution with the features of interest could indicate a higher risk of coastal hazards. Therefore, some MPA management actions could be considered, like updating vulnerability assessment and construction of coastal protection structures. The significant influence of particular marine tourism features should also be viewed as a warning to prevent the depletion of those resources by mass utilisation. Management could revise zoning plans and update the recreation fees to prevent resource depletion. Situated mainly on the eastern coast of Gili Trawangan, high-priced properties could be included in the future cost–benefit analyses to provide quantitatively informed recommendations, such as beach nourishment to prevent erosion.

The positive influence of MPA’s other zones (less strict zones) on CES well-being could be considered in MPA management by shifting the current core zones seaward and allocating more zones with less strict restrictions. Additionally, spatial planning for land use could be updated as the public amenities reportedly affected Gili Matra residents’ spiritual value and place identity. However, CES eudaemonic well-being values are often subjective and intangible. MPA managers must understand that they could only influence such well-being outputs passively.

## Figures and Tables

**Table 1:** Statistical summary for model HR1 of residential property prices in Gili Matra Islands.

Variable	Description	Min.	Max.	Mean	Std. Dev.
Distance Beach spots	Distance (m) from property to the nearest beach spot	75.21	1200.19	536.34	248.66
Visibility Beach spots	0: no beach spot visible; 1: at least 1 beach spot visible	0	1	0.04	0.19
Distance Coastline	Distance (m) from property to the nearest coastline	22.93	810.49	309.11	146.50
Visibility Coastline	0: no coastline visible; 1: at least 1 coastline visible	0	1	0.22	0.42
Distance Coastline Sunset	Distance (m) from property to nearest sunset coastline	22.93	1180.49	661.58	287.44
Visibility Coastline Sunset	0: no sunset coastline visible; 1: at least 1 visible	0	1	0.13	0.34
Distance Dive spot	Distance (m) from property to nearest dive spot	240.08	1051.83	592.04	145.02
Visibility Dive spot	0: no dive spot visible; 1: at least 1 dive spot visible	0	1	0.13	0.34
Distance Core zone	Distance (m) from property to nearest MPA core zone	60.49	1620.35	990.68	332.73
Visibility Core zone	0: no core zone visible; 1: at least 1 core zone visible	0	1	0.05	0.21
Distance Other zones	Distance (m) from property to nearest other zones	17.99	813.19	308.43	142.59
Visibility Other zones	0: no other zone visible; 1: at least 1 other zone visible	0	1	0.33	0.47
Distance Facilities	Distance (m) from property to nearest public amenities	20.73	1252.66	288.51	303.01
Visibility Facilities	0: no amenities visible; 1: at least 1 amenity visible	0	1	0.48	0.50
Distance Road	Distance (m) from property to nearest road	0.97	171.86	28.09	27.07
Visibility Road	0: no road visible; 1: at least 1 road visible	1	1	1	0
Residential Properties Prices	in Indonesian Rupiah (IDR) million per are (100 m^2^)	75.00	1000.00	228.87	158.42

**Table 2:** Distribution of case examples in each quartile of residential property prices.

Quartile	Case Example	Location
1ST QUARTILE (<25%, <IDR 133.3 MILLION PER **ARE**)	IDR 75 million per are. Gili Meno. 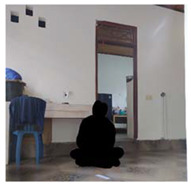	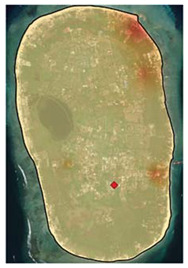
2ND QUARTILE (25–50%, IDR 133.33–175 MILLION PER **ARE**)	IDR 170 million per are. Gili Air. 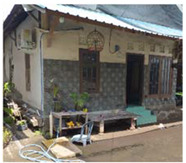	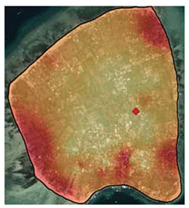
3RD QUARTILE (50–75%, IDR 175–266.67 MILLION PER **ARE**)	IDR 263 million per are. Gili Air. 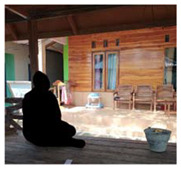	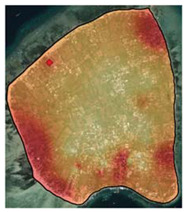
4TH QUARTILE (75–100%, IDR 266.67–1000.00 MILLION PER **ARE**)	IDR 1000 million per are. Gili Trawangan. 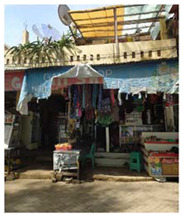	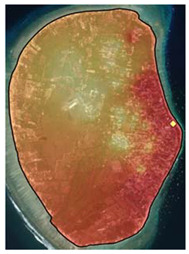

**Table 3:** Statistical summary for model HT1 of tourism accommodation property prices.

Variable	Description	Min.	Max.	Mean	Std. Dev.
Distance Beach spots	Distance from the property to the nearest beach spot	112.40	1053.84	474.91	248.44
Visibility Beach spots	0: no beach spot visible; 1: at least 1 beach spot visible	0	1	0.07	0.25
Distance Coastline	Distance from the property to the nearest coastline	23.60	819.65	258.93	208.16
Visibility Coastline	0: no coastline visible; 1: at least 1 coastline visible	0	1	0.33	0.47
Distance Coastline Sunset	Distance from the property to the nearest sunset coastline	23.60	1025.43	481.11	310.41
Visibility Coastline Sunset	0: no sunset coastline visible; 1: at least 1 visible	0	1	0.26	0.44
Distance Dive spot	Distance from the property to the nearest dive spot	165.71	1072.44	551.93	221.63
Visibility Dive spot	0: no dive spot visible; 1: at least 1 dive spot visible	0	1	0.37	0.49
Distance Core zone	Distance from the property to the nearest MPA core zone	35.78	1585.74	969.14	380.69
Visibility Core zone	0: no core zone visible; 1: at least 1 core zone visible	0	1	0.13	0.34
Distance Other zones	Distance from the property to nearest other zones	17.14	849.42	264.88	207.41
Visibility Other zones	0: no other zone visible; 1: at least 1 other zone visible	0	1	0.48	0.51
Distance Facilities	Distance from the property to nearest public amenities	41.97	1694.95	750.77	490.14
Visibility Facilities	0: no amenities visible; 1: at least 1 amenity visible	0	1	0.13	0.34
Distance Road	Distance from the property to the nearest road	5.87	141.44	41.29	35.88
Visibility Road	0: no road visible; 1: at least 1 road visible	0	1	0.96	0.21
Bedroom Number	Bedroom number	1	60	10.41	12.03
Tourism Accommodation Properties Prices	Tourism accommodation properties prices in Indonesian Rupiah (IDR) million	650.00	266,864.00	18,341.98	41,342.32

**Table 4:** Summary of multiple regression results between CES well-being dimensions and marine tourism and protection features.

Independent Variables	SKILL		MEM		SOC		SPIR		THEU		PLID		ENG	
	Coef (SD.)	Sig.	Coef (SD.)	Sig.	Coef (SD.)	Sig.	Coef (SD.)	Sig.	Coef (SD.)	Sig.	Coef (SD.)	Sig.	Coef (SD.)	Sig.
Constant	3.914 (0.320)	0.000	5.766 (0.473)	0.000	5.975 (0.379)	0.000	7.434 (0.497)	0.000	5.463 (0.416)	0.000	5.728 (0.477)	0.000	4.042 (0.284)	0.000
Distance to the nearest beach spot	0.000 (0.000)	0.450	0.000 (0.000)	0.223	0.000 (0.000)	0.113	−0.001 (0.000)	**0.009**	0.000 (0.000)	0.438	0.000 (0.000)	0.478	0.000 (0.000)	0.312
Visibility of at least 1 beach spot	0.164 (0.265)	0.537	−0.085 (0.391)	0.828	−0.227 (0.314)	0.472	−0.257 (0.411)	0.533	−0.163 (0.344)	0.636	−0.082 (0.395)	0.837	0.158 (0.235)	0.503
Distance to the nearest coastline	0.002 (0.001)	**0.005**	0.002 (0.001)	**0.012**	0.002 (0.001)	**0.003**	0.002 (0.001)	**0.021**	0.001 (0.001)	**0.050**	0.003 (0.001)	**0.004**	0.002 (0.001)	**0.004**
Visibility of coastline	0.132 (0.155)	0.399	0.239 (0.229)	0.299	0.315 (0.184)	0.090	0.481 (0.241)	**0.048**	0.308 (0.202)	0.129	0.265 (0.232)	0.254	0.133 (0.138)	0.337
Distance to nearest sunset coastline	0.000 (0.000)	0.809	0.000 (0.000)	0.862	0.000 (0.000)	0.216	0.001 (0.000)	0.075	0.000 (0.000)	0.640	0.000 (0.000)	0.127	0.000 (0.000)	0.254
Visibility of coastline facing the sunset	−0.257 (0.225)	0.256	−0.394 (0.332)	0.238	−0.387 (0.266)	0.149	−0.225 (0.349)	0.521	−0.313 (0.292)	0.286	−0.656 (0.335)	0.053	−0.263 (0.200)	0.190
Distance to the nearest dive spot	0.000 (0.000)	0.451	0.000 (0.001)	0.935	0.000 (0.000)	0.752	0.000 (0.001)	0.473	0.000 (0.001)	0.440	0.000 (0.001)	0.458	0.000 (0.000)	0.949
Visibility of at least 1 dive spot	0.009 (0.204)	0.965	−0.074 (0.301)	0.807	0.010 (0.241)	0.968	−0.142 (0.316)	0.653	−0.158 (0.264)	0.552	0.376 (0.303)	0.218	0.151 (0.181)	0.406
Distance to nearest MPA core zone	0.000 (0.000)	0.324	0.000 (0.000)	0.167	0.000 (0.000)	**0.041**	−0.001 (0.000)	**0.005**	0.000 (0.000)	0.278	0.000 (0.000)	0.144	0.000 (0.000)	0.248
Visibility of at least 1 core zone	−0.010 (0.254)	0.970	0.324 (0.375)	0.390	0.277 (0.301)	0.360	0.274 (0.395)	0.490	0.261 (0.330)	0.431	0.044 (0.379)	0.907	−0.182 (0.226)	0.423
Distance to nearest other zones	−0.002 (0.001)	**0.002**	−0.002 (0.001)	**0.018**	−0.002 (0.001)	**0.003**	−0.002 (0.001)	**0.031**	−0.002 (0.001)	**0.029**	−0.003 (0.001)	**0.006**	−0.002 (0.001)	**0.002**
Visibility of at least 1 other zones	0.024 (0.110)	0.828	0.102 (0.163)	0.531	−0.079 (0.131)	0.545	−0.193 (0.171)	0.261	0.023 (0.143)	0.874	−0.097 (0.164)	0.557	−0.048 (0.098)	0.628
Distance to nearest public amenities	0.000 (0.000)	0.075	0.000 (0.000)	0.186	0.000 (0.000)	**0.017**	0.001 (0.000)	**0.026**	0.000 (0.000)	0.136	0.001 (0.000)	**0.028**	0.000 (0.000)	0.061
Visibility of at least 1 amenity	0.075 (0.083)	0.369	0.094 (0.122)	0.446	0.168 (0.098)	0.090	0.130 (0.129)	0.315	0.084 (0.108)	0.436	0.270 (0.124)	**0.031**	0.086 (0.074)	0.243
Distance to the nearest road	0.000 (0.001)	0.993	−0.001 (0.002)	0.662	−0.001 (0.002)	0.453	−0.003 (0.002)	0.235	−0.001 (0.002)	0.523	0.000 (0.002)	0.851	0.000 (0.001)	0.885
R Square	0.164		0.133		0.151		0.155		0.148		0.128		0.123	

SKILL: Skill and challenge dimension; MEM: Memory/Transformative value; SOC: Social bonds; SPIR: Spiritual value; THEU: Therapeutic value; PLID: Place identity; ENG: Engagement and interaction with nature. Bold typed values indicated high significance.

## Data Availability

Digital Elevation Model (DEM) data can be derived from United States Geological Survey (USGS) online database, which is openly available. Questionnaires and analysed results are contained within the Supplementary Materials.

## Supplementary Materials

The following supporting information can be downloaded at: https://www.mdpi.com/article/10.3390/ijerph191912078/s1, Questionnaire S1: Hedonic and Eudaemonic Questionnaire; Equation (S1): Residential Property Prices Gili Matra (model HR1); Table S1: T-test result from standard multiple regression analysis for model HR1; Table S2: ANOVA test result from standard multiple regression analysis for model HR1; Equation (S2): Residential Property Prices Gili Trawangan (model HR2); Table S3: T-test result from standard multiple regression analysis for model HR2; Table S4: ANOVA test result from standard multiple regression analysis for model HR2; Equation (S3): Residential Property Prices Gili Air (model HR3); Table S5: T-test result from standard multiple regression analysis for model HR3; Table S6: ANOVA test result from standard multiple regression analysis for model HR3; Equation (S4): Residential Property Prices Gili Meno (model HR4); Table S7: T-test result from standard multiple regression analysis for model HR4; Table S8: ANOVA test result from standard multiple regression analysis for model HR4; Equation (S5): Tourism Accommodation Property Prices, after elimination (model HT1); Table S9: T-test result from standard multiple regression analysis for model HT1, after elimination; Table S10: ANOVA test result from standard multiple regression analysis for model HT1, after elimination; Equation (S6): “Skill” factor scores for Response #1; Table S11: T-test result from standard multiple regression analysis for model R1 “Skill”; Table S12: ANOVA test result from standard multiple regression analysis for model R1 “Skill”; Table S13: T-test result from standard multiple regression analysis for model R2 “Engagement”; Table S14: ANOVA test result from standard multiple regression analysis for model R2 “Engagement”; Table S15: T-test result from standard multiple regression analysis for model R3 “Memory”; Table S16: ANOVA test result from standard multiple regression analysis for model R3 “Memory”; Table S17: T-test result from standard multiple regression analysis for model R4 “Place Identity”; Table S18: ANOVA test result from standard multiple regression analysis for model R4 “Place Identity”; Table S19: T-test result from standard multiple regression analysis for model R5 “Social”; Table S20: ANOVA test result from standard multiple regression analysis for model R5 “Social”; Table S21: T-test result from standard multiple regression analysis for model R6 “Spiritual”; Table S22: ANOVA test result from standard multiple regression analysis for model R6 “Spiritual”; Table S23: T-test result from standard multiple regression analysis for model R7 “Therapeutic”; Table S24: ANOVA test result from standard multiple regression analysis for model R7 “Therapeutic”; Table S25: Communalities values for Model E1; Table S26: The goodness of fit indicators used for Model E1; Table S27: Factor score weight produced for Model E1 Gili Matra.

## Appendix A

**Table A1:** The adapted indicator and factor used in the well-being instrument [4].

No.	Indicator Question	Well-Being Factors Associated with CES
1	I feel more connected to my environment due to the natural places near where I live.	Engagement and interaction with nature (Eng)
2	I have learned more about the environment by visiting natural places near my residence.	
3	I believe I can contribute by taking care of natural places adjacent to my residence.	
4	The beauty of natural places near my residence has touched me.	
5	I get inspiration from natural places near my residence.	
6	I feel like the natural places near my residence are almost part of me.	Place identity (Plid)
7	I feel like the natural places near my residence belong to me.	
8	When I am away from my home for a long time, I miss those natural places.	
9	My head is relaxed when I am in the natural places near my residence.	Therapeutic value (Theu)
10	I feel healthier when I am in the natural places near my residence.	
11	I feel freedom when I am in the natural places near my residence.	
12	I believe there is something greater than me when I am in natural places near my residence.	Spiritual value (Spir)
13	I believe that I have the meaning of life given by natural places near where I live.	
14	I have deeper experience in my life because of the natural places near my residence.	
15	I have bonds with the natural places near where I live.	Social bonds (Soc)
16	I am more connected to people because of the natural places near my residence.	
17	I have a better sense of community because of the natural places near my residence.	
18	I have plenty of great memories of the natural places near where I live.	Memory/Transformative value (Mem)
19	I have changed because of the natural places near where I live.	
20	I remember the time I had spent at the natural places near my residence.	
21	I have chances to challenge myself because of the natural places near my home.	Skill and challenge (Skill)
22	I have chances to test my skills and abilities because of the natural places near my residence.	
23	I have chances to enjoy myself in the natural places near my residence.	
