# Supplementary material for: Assessing the Impact of Marine Tourism and Protection on Cultural Ecosystem Services Using Integrated Approach: A Case Study of Gili Matra Islands
Source: Int J Environ Res Public Health. 2022 Sep 24;19(19):12078. doi: 10.3390/ijerph191912078 (PMC9564878; doi:10.3390/ijerph191912078)
Supplement: Supplementary file 1 [file ijerph-19-12078-s001.zip › ijerph-1874523-supplementary.pdf]

Supplementary Materials

Assessing the Impact of Marine Tourism and Marine Protection on Cultural Ecosystem Services Using an Integrated Hedonic and Eudaemonic Approach: A case study of Gili Matra Islands

Questionnaire S1.

Hedonic and Eudaemonic Questionnaire

Part A

1	**Question**: What is your name?<br>Answer:
2	**Question**: What is your age?<br>Answer:
3	**Question**: What is your gender?<br>Answer:
4	**Question**: What do you do for a living?<br>Answer:

Part B

Below are the questions for **property where you live** (such as a house or renthouse) and for other **properties you belong if any** (such as another house, hotel, cottage, vacant land, shop etc.).
Please fill in the answers as true as possible according to your knowledge.

1	**For the property where you live in** (house or renthouse)
	A. Location<br>1. Where are the address and the street name? (If able, you can include the coordinate from google maps).<br>Answer:<br>2. What is the nearby public/private facility? (Such as hotel, cottage, government office, market etc.)<br>Answer:
	B. Estimated price<br>1. How much do you estimate is the price of this property? (Please use a total price and also price per square meter or hectare)<br>Answer:
2	**For other properties you belong, if any** (can be more than 1, such as another house, hotel/cottage, vacant land, shop, market and so forth)
	A. Location<br>1. Where are the address and the street name? (If able, you can include the coordinate).<br>Answer:<br>2. What is the nearby public/private facility? (Such as hotel, cottage, government office, market etc.) Or you can use your property name such as "Hotel …" or "Shop …"<br>Answer:
	B. Estimated price<br>1. How much do you estimate is the price of this property? (Please use a total price and also price per square meter or hectare)<br>Answer:

Part C

Questions below are how you're feeling about **natural places nearby your residence**. Please circle the number of what you prefer for each statement.

No	Indicator question						
1	**I feel more connected to my environment due to the natural places near where I live.**						
	1<br>Strongly	2<br>Disagree	3<br>Somewhat	4<br>Neutral	5<br>Somewhat	6<br>Agree	7<br>Strongly

2	**I have learned more about the environment by visiting natural places near my residence.**						
	1<br>Strongly	2<br>Disagree	3<br>Somewhat	4<br>Neutral	5<br>Somewhat	6<br>Agree	7<br>Strongly
3	**I believe I can contribute by taking care of natural places adjacent to my residence.**						
	1<br>Strongly	2<br>Disagree	3<br>Somewhat	4<br>Neutral	5<br>Somewhat	6<br>Agree	7<br>Strongly
4	**The beauty of natural places near my residence has touched me.**						
	1<br>Strongly	2<br>Disagree	3<br>Somewhat	4<br>Neutral	5<br>Somewhat	6<br>Agree	7<br>Strongly
5	**I get inspiration from natural places near my residence.**						
	1<br>Strongly	2<br>Disagree	3<br>Somewhat	4<br>Neutral	5<br>Somewhat	6<br>Agree	7<br>Strongly
6	**I feel like the natural places near my residence are almost part of me.**						
	1<br>Strongly	2<br>Disagree	3<br>Somewhat	4<br>Neutral	5<br>Somewhat	6<br>Agree	7<br>Strongly
7	**I feel like the natural places near my residence belong to me.**						
	1<br>Strongly	2<br>Disagree	3<br>Somewhat	4<br>Neutral	5<br>Somewhat	6<br>Agree	7<br>Strongly
8	**When I am away from my home for a long time, I miss those natural places.**						
	1<br>Strongly	2<br>Disagree	3<br>Somewhat	4<br>Neutral	5<br>Somewhat	6<br>Agree	7<br>Strongly
9	**My head is relaxed when I am in the natural places near my residence.**						
	1<br>Strongly	2<br>Disagree	3<br>Somewhat	4<br>Neutral	5<br>Somewhat	6<br>Agree	7<br>Strongly
10	**I feel healthier when I am in the natural places near my residence.**						
	1<br>Strongly	2<br>Disagree	3<br>Somewhat	4<br>Neutral	5<br>Somewhat	6<br>Agree	7<br>Strongly
11	**I feel freedom when I am in the natural places near my residence.**						
	1<br>Strongly	2<br>Disagree	3<br>Somewhat	4<br>Neutral	5<br>Somewhat	6<br>Agree	7<br>Strongly
12	**I believe there is something greater than me when I am in natural places near my residence.**						
	1<br>Strongly	2<br>Disagree	3<br>Somewhat	4<br>Neutral	5<br>Somewhat	6<br>Agree	7<br>Strongly
13	**I believe that I have the meaning of life given by natural places near where I live.**						
	1<br>Strongly	2<br>Disagree	3<br>Somewhat	4<br>Neutral	5<br>Somewhat	6<br>Agree	7<br>Strongly
14	**I have deeper experience in my life because of the natural places near my residence.**						
	1<br>Strongly	2<br>Disagree	3<br>Somewhat	4<br>Neutral	5<br>Somewhat	6<br>Agree	7<br>Strongly
15	**I have bonds with the natural places near where I live.**						
	1<br>Strongly	2<br>Disagree	3<br>Somewhat	4<br>Neutral	5<br>Somewhat	6<br>Agree	7<br>Strongly

16	**I am more connected to people because of the natural places near my residence.**						
	1<br>Strongly	2<br>Disagree	3<br>Somewhat	4<br>Neutral	5<br>Somewhat	6<br>Agree	7<br>Strongly
17	**I have a better sense of community because of the natural places near my residence.**						
	1<br>Strongly	2<br>Disagree	3<br>Somewhat	4<br>Neutral	5<br>Somewhat	6<br>Agree	7<br>Strongly
18	**I have plenty of great memories of the natural places near where I live.**						
	1<br>Strongly	2<br>Disagree	3<br>Somewhat	4<br>Neutral	5<br>Somewhat	6<br>Agree	7<br>Strongly
19	**I have changed because of the natural places near where I live.**						
	1<br>Strongly	2<br>Disagree	3<br>Somewhat	4<br>Neutral	5<br>Somewhat	6<br>Agree	7<br>Strongly
20	**I remember the time I had spent at the natural places near my residence.**						
	1<br>Strongly	2<br>Disagree	3<br>Somewhat	4<br>Neutral	5<br>Somewhat	6<br>Agree	7<br>Strongly
21	**I have chances to challenge myself because of the natural places near my home.**						
	1<br>Strongly	2<br>Disagree	3<br>Somewhat	4<br>Neutral	5<br>Somewhat	6<br>Agree	7<br>Strongly
22	**I have chances to test my skills and abilities because of the natural places near my residence.**						
	1<br>Strongly	2<br>Disagree	3<br>Somewhat	4<br>Neutral	5<br>Somewhat	6<br>Agree	7<br>Strongly
23	**I have chances to enjoy myself in the natural places near my residence.**						
	1<br>Strongly	2<br>Disagree	3<br>Somewhat	4<br>Neutral	5<br>Somewhat	6<br>Agree	7<br>Strongly

Equation S1

Residential Property Prices Gili Matra (model HR1) =
415.027 - 0.128 Dist_Beachspot + 57.890 Vis_Beachspot - 0.697 Dist_Coastline + 27.959 Vis_Coastline + 0.093 Dist_CoastlineSunset + 143.952 Vis_CoastlineSunset + 0.120 Dist_Divespot - 43.662 Vis_Divespot - 0.004 Dist_Corezone + 5.978 Vis_Corezone - 0.077 Dist_Otherzone - 40.180 Vis_Otherzone + 0.037 Dist_Facilities - 17.593 Vis_Facilities - 0.640 Dist_Road

Table S1. T-test result from standard multiple regression analysis for model HR1

	Unstandardised Coefficients		Standard. Coef.	t	Sig.
Variable	B	Std. Error	Beta		
(Constant)	415.027	87.117		4.764	.000
Distance-Beachspot	-.128	.049	-.200	-2.629	**.010**
Visibility-Beachspot	57.890	72.392	.070	.800	.426
Distance-Coastline	-.697	.157	-.645	-4.443	**.000**
Visibility-Coastline	27.959	42.429	.074	.659	.511
Distance-Coastline Sunset	.093	.049	.169	1.886	.062
Visibility-Coastline Sunset	143.952	61.375	.307	2.345	**.021**
Distance-Divespot	.120	.106	.110	1.134	.259
Visibility-Divespot	-43.662	55.644	-.093	-.785	.434

Distance-Corezone	-.004	.046	-.008	-.082	.935
Visibility-Corezone	5.978	69.499	.008	.086	.932
Distance-Otherzone	-.077	.166	-.070	-.466	.642
Visibility-Otherzone	-40.180	30.134	-.120	-1.333	.185
Distance-Facilities	.037	.043	.071	.860	.391
Visibility-Facilities	-17.593	22.172	-.056	-.793	.429
Distance-Road	-.640	.407	-.109	-1.571	.119

Table S2. ANOVA test result from standard multiple regression analysis for model HR1

ANOVA[a]

Model		Sum of Squares	df	Mean Square	F	Sig.
1	Regression	2092022.942	15	139468.196	13.700	.000[b]
	Residual	1170694.326	115	10179.951		
	Total	3262717.268	130			

a. Dependent Variable: Primary_Residence_Price_upd

b. Predictors: (Constant), Dist_Road, Vis_Beachspot, Dist_Beachspot, Dist_Divespot, Vis_Facilities, Vis_Divespot, Dist_CoastlineWN, Dist_Facilities, Vis_Otherzone, Dist_Corezone, Dist_Coastline, Vis_Corezone, Vis_Coastline, Vis_CoastlineWN, Dist_Otherzone

Equation S2

Residential Property Prices Gili Trawangan (model HR2) =
851.209 + 0.491 Dist_Beachspot - 1.011 Dist_Coastline + 103.145 Vis_Coastline - 0.478 Dist_CoastlineSunset + 42.638 Vis_CoastlineSunset + 0.399 Dist_Divespot - 22.609 Vis_Divespot - 0.032 Dist_Corezone + 77.543 Vis_Corezone - 0.051 Dist_Otherzone - 36.697 Vis_Otherzone - 0.464 Dist_Facilities - 12.699 Vis_Facilities - 0.661 Dist_Road

Table S3. T-test result from standard multiple regression analysis for model HR2.

	Unstandardised Coefficients		Standard. Coef.	t	Sig.
Variable	B	Std. Error	Beta		
(Constant)	851.209	271.253		3.138	.003
Distance-Beachspot	.491	.422	.489	1.164	.252
Distance-Coastline	-1.011	.475	-.980	-2.128	.040
Visibility-Coastline	103.145	84.026	.271	1.228	.228
Distance-Coastline Sunset	-.478	.259	-.656	-1.843	.074
Visibility-Coastline Sunset	42.638	120.067	.101	.355	.725
Distance-Divespot	.399	.266	.341	1.503	.141
Visibility-Divespot	-22.609	90.147	-.050	-.251	.803
Distance-Corezone	-.032	.157	-.041	-.203	.841
Visibility-Corezone	77.543	130.059	.062	.596	.555
Distance-Otherzone	-.051	.299	-.049	-.169	.867
Visibility-Otherzone	-36.697	47.285	-.105	-.776	.443
Distance-Facilities	-.464	.215	-.946	-2.164	.037
Visibility-Facilities	-12.699	41.162	-.037	-.309	.759
Distance-Road	-.661	.752	-.136	-.878	.386

Table S4. ANOVA test result from standard multiple regression analysis for model HR2.

ANOVA[a]

Model		Sum of Squares	df	Mean Square	F	Sig.
1	Regression	1143489.805	14	81677.843	7.475	.000[b]
	Residual	393363.753	36	10926.771		
	Total	1536853.558	50			

a. Dependent Variable: Primary_Residence_Price

b. Predictors: (Constant), Dist_Road, Dist_Divespot, Vis_Divespot, Vis_Corezone, Vis_Facilities, Dist_CoastlineWN, Dist_Corezone, Vis_Otherzone, Vis_Coastline, Dist_Beachspot, Dist_Otherzone, Vis_CoastlineWN, Dist_Facilities, Dist_Coastline

Equation S3

Residential Property Prices Gili Air (model HR3) =
1480.448 - 0.467 Dist_Beachspot - 159.158 Vis_Beachspot - 0.604 Dist_Coastline - 136.556 Vis_Coastline + 0.032 Dist_CoastlineSunset - 1.003 Dist_Divespot - 68.591 Vis_Divespot - 0.454 Dist_Corezone + 269.768 Vis_Corezone + 0.630 Dist_Otherzone + 12.005 Vis_Otherzone + 0.078 Dist_Facilities - 32.917 Vis_Facilities - 2.004 Dist_Road

Table S5. T-test result from standard multiple regression analysis for model HR3.

	Unstandardised Coefficients		Standard. Coef.	t	Sig.
Variable	B	Std. Error	Beta		
(Constant)	1480.448	294.343		5.030	.000
Distance-Beachspot	-.467	.176	-.627	-2.658	**.012**
Visibility-Beachspot	-159.158	157.988	-.269	-1.007	.321
Distance-Coastline	-.604	1.183	-.498	-.510	.613
Visibility-Coastline	-136.556	69.130	-.327	-1.975	.056
Distance-Coastline Sunset	.032	.232	.042	.140	.889
Distance-Divespot	-1.003	.317	-1.007	-3.163	**.003**
Visibility-Divespot	-68.591	163.537	-.116	-.419	.677
Distance-Corezone	-.454	.193	-.803	-2.349	**.025**
Visibility-Corezone	269.768	212.862	.505	1.267	.213
Distance-Otherzone	.630	1.220	.512	.516	.609
Visibility-Otherzone	12.005	52.858	.033	.227	.822
Distance-Facilities	.078	.308	.104	.253	.802
Visibility-Facilities	-32.917	36.258	-.103	-.908	.370
Distance-Road	-2.004	.823	-.258	-2.434	**.020**

Table S6. ANOVA test result from standard multiple regression analysis for model HR3.

ANOVA[a]

Model		Sum of Squares	df	Mean Square	F	Sig.
1	Regression	936218.997	14	66872.785	6.721	.000[b]
	Residual	348254.940	35	9950.141		
	Total	1284473.937	49			

a. Dependent Variable: Primary_Residence_Price

b. Predictors: (Constant), Dist_Road, Dist_CoastlineWN, Vis_Facilities, Dist_Divespot, Vis_Coastline, Dist_Corezone, Vis_Beachspot, Vis_Otherzone, Dist_Beachspot, Vis_Divespot, Dist_Coastline, Vis_Corezone, Dist_Facilities, Dist_Otherzone

Equation S4

Residential Property Prices Gili Meno (model HR4) =
402.822 + 0.103 Dist_Beachspot + 86.517 Vis_Beachspot - 0.271 Dist_Coastline - 29.233 Vis_Coastline - 0.002 Dist_CoastlineSunset + 41.887 Vis_CoastlineSunset - 0.132 Dist_Divespot + 7.987 Vis_Divespot - 0.140 Dist_Corezone + 0.019 Dist_Otherzone - 12.080 Vis_Otherzone - 0.086 Dist_Facilities - 8.603 Vis_Facilities + 0.176 Dist_Road

Table S7. T-test result from standard multiple regression analysis for model HR4.

	Unstandardised Coefficients		Standard. Coef.	t	Sig.
Variable	B	Std. Error	Beta		
(Constant)	402.822	113.831		3.539	.003
Distance-Beachspot	.103	.125	.535	.822	.424
Visibility-Beachspot	86.517	43.185	.304	2.003	.064
Distance-Coastline	-.271	.115	-.604	-2.362	**.032**
Visibility-Coastline	-29.233	32.419	-.214	-.902	.381
Distance-Coastline Sunset	-.002	.097	-.005	-.018	.986
Visibility-Coastline Sunset	41.887	31.327	.205	1.337	.201
Distance-Divespot	-.132	.123	-.245	-1.071	.301
Visibility-Divespot	7.987	32.162	.053	.248	.807
Distance-Corezone	-.140	.065	-.441	-2.162	**.047**
Distance-Otherzone	.019	.093	.037	.209	.838
Visibility-Otherzone	-12.080	28.561	-.100	-.423	.678
Distance-Facilities	-.086	.096	-.462	-.899	.383
Visibility-Facilities	-8.603	14.524	-.079	-.592	.562
Distance-Road	.176	.443	.053	.398	.696

Table S8. ANOVA test result from standard multiple regression analysis for model HR4.

ANOVA[a]

Model		Sum of Squares	df	Mean Square	F	Sig.
1	Regression	72492.333	14	5178.024	7.159	.000[b]
	Residual	10849.348	15	723.290		
	Total	83341.681	29			

a. Dependent Variable: Primary_Residence_Price

b. Predictors: (Constant), Dist_Road, Dist_Divespot, Dist_Facilities, Vis_Divespot, Vis_Beachspot, Dist_Corezone, Vis_Facilities, Vis_CoastlineWN, Dist_CoastlineWN, Dist_Otherzone, Vis_Coastline, Dist_Coastline, Vis_Otherzone, Dist_Beachspot

Equation S5

Tourism Accommodation Property Prices, after elimination (model HT1) =

23,953.45 + 523.915 Dist_Coastline - 18,222.128 Vis_Coastline - 43.686 Dist_CoastlineSunset - 20,949.886 Vis_CoastlineSunset + 29,446.455 Vis_Corezone - 495.421 Dist_Otherzone - 30,042.832 Vis_Otherzone - 16.355 Dist_Facilities - 11,734.527 Vis_Facilities - 164.494 Dist_Road + 2,379.588 Bedroom

Table S9. T-test result from standard multiple regression analysis for model HT1, after elimination.

	Unstandardised Coefficients		Standard. Coef.	t	Sig.
Variable	B	Std. Error	Beta		
(Constant)	23,953.45	14,389.97		1.665	0.105
Distance-Coastline	523.915	262.281	2.638	1.998	**0.054**
Visibility-Coastline	-18,222.128	16,114.06	-0.209	-1.131	0.266
Distance-Coastline Sunset	-43.686	17.153	-0.328	-2.547	**0.016**
Visibility-Coastline Sunset	-20,949.886	15,097.42	-0.225	-1.388	0.174
Visibility-Corezone	29,446.455	12,314.04	0.243	2.391	**0.022**
Distance-Otherzone	-495.421	264.786	-2.485	-1.871	0.070
Visibility-Otherzone	30,042.832	12,156.42	0.367	2.471	**0.019**
Distance-Facilities	-16.355	11.46	-0.194	-1.427	0.163
Visibility-Facilities	-11,734.527	11,195.24	-0.097	-1.048	0.302
Distance-Road	-164.494	93.576	-0.143	-1.758	0.088
Bedroom number	2,379.588	320.035	0.692	7.435	**0.000**

Table S10. ANOVA test result from standard multiple regression analysis for model HT1, after elimination.

ANOVA[a]

Model		Sum of Squares	df	Mean Square	F	Sig.
1	Regression	63520909276.016	17	3736524075.060	7.812	.000[b]
	Residual	13392527474.962	28	478304552.677		
	Total	76913436750.978	45			

a. Dependent Variable: Secondary_Accom_Price

b. Predictors: (Constant), Bedroom, Dist_CoastlineWN, Vis_Coastline, Dist_Road, Dist_Corezone, Vis_Facilities, Vis_Road, Dist_Divespot, Dist_Beachspot, Vis_Beachspot, Dist_Facilities, Vis_Corezone, Vis_Divespot, Vis_CoastlineWN, Vis_Otherzone, Dist_Coastline, Dist_Otherzone

Equation S6

"Skill" factor scores for Response #1 =
Eng4 * .02 + Skill21 * .153 + Skill22 * .104 + Skill23 * .078 + Mem18 * .047 + Mem19 * .035 + Mem20 * .021 + Soc15 * .032 + Soc16 * .012 + Spir12 * -0.003 + Spir13 * -0.007 + Spir14 * -0.022 + Theu9 * .029 + Theu10 * .036 + Theu11 * 0.011 + Plid6 * 0.001 + Plid7 * 0 + Eng1 * .032 + Eng2 * .041

Table S11. T-test result from standard multiple regression analysis for model R1 "Skill"

	Unstandardised Coefficients		Standard. Coef.	t	Sig.
Variable	B	Std. Error	Beta		

(Constant)	3.914	.320		12.219	.000
Dist_Beachspot	.000	.000	-.088	-.758	.450
Vis_Beachspot	.164	.265	.084	.619	.537
Dist_Coastline	.002	.001	.645	2.865	.005
Vis_Coastline	.132	.155	.146	.847	.399
Dist_CoastlineWN	4.389E-5	.000	.033	.243	.809
Vis_CoastlineWN	-.257	.225	-.231	-1.142	.256
Dist_Divespot	.000	.000	.115	.756	.451
Vis_Divespot	.009	.204	.008	.044	.965
Dist_Corezone	.000	.000	-.149	-.991	.324
Vis_Corezone	-.010	.254	-.005	-.038	.970
Dist_Otherzone	-.002	.001	-.737	-3.189	.002
Vis_Otherzone	.024	.110	.030	.218	.828
Dist_Facilities	.000	.000	.232	1.798	.075
Vis_Facilities	.075	.083	.099	.902	.369
Dist_Road	-1.373E-5	.001	-.001	-.009	.993

Table S12. ANOVA test result from standard multiple regression analysis for model R1 "Skill"

ANOVA[a]

Model		Sum of Squares	df	Mean Square	F	Sig.
1	Regression	2.984	15	.199	1.460	.133[b]
	Residual	15.262	112	.136		
	Total	18.246	127			

a. Dependent Variable: SKILL

b. Predictors: (Constant), Dist_Road, Vis_Beachspot, Dist_Beachspot, Dist_Divespot, Vis_Facilities, Vis_Divespot, Dist_CoastlineWN, Dist_Facilities, Vis_Otherzone, Dist_Corezone, Vis_Corezone, Dist_Coastline, Vis_Coastline, Vis_CoastlineWN, Dist_Otherzone

Table S13. T-test result from standard multiple regression analysis for model R2 "Engagement"

	Unstandardised Coefficients		Standard. Coef.	t	Sig.
Variable	B	Std. Error	Beta		
(Constant)	4.042	.284		14.214	.000
Dist_Beachspot	.000	.000	-.121	-1.015	.312
Vis_Beachspot	.158	.235	.094	.672	.503
Dist_Coastline	.002	.001	.680	2.951	.004
Vis_Coastline	.133	.138	.170	.964	.337
Dist_CoastlineWN	.000	.000	.162	1.146	.254
Vis_CoastlineWN	-.263	.200	-.273	-1.318	.190
Dist_Divespot	2.220E-5	.000	.010	.063	.949
Vis_Divespot	.151	.181	.156	.834	.406
Dist_Corezone	.000	.000	-.179	-1.162	.248
Vis_Corezone	-.182	.226	-.117	-.805	.423
Dist_Otherzone	-.002	.001	-.737	-3.115	.002
Vis_Otherzone	-.048	.098	-.069	-.486	.628

Dist_Facilities	.000	.000	.250	1.895	.061
Vis_Facilities	.086	.074	.132	1.174	.243
Dist_Road	.000	.001	-.016	-.145	.885

Table S14. ANOVA test result from standard multiple regression analysis for model R2 "Engagement"

ANOVA[a]

Model		Sum of Squares	df	Mean Square	F	Sig.
1	Regression	1.692	15	.113	1.050	.411[b]
	Residual	12.029	112	.107		
	Total	13.721	127			

a. Dependent Variable: ENG

b. Predictors: (Constant), Dist_Road, Vis_Beachspot, Dist_Beachspot, Dist_Divespot, Vis_Facilities, Vis_Divespot, Dist_CoastlineWN, Dist_Facilities, Vis_Otherzone, Dist_Corezone, Vis_Corezone, Dist_Coastline, Vis_Coastline, Vis_CoastlineWN, Dist_Otherzone

Table S15. T-test result from standard multiple regression analysis for model R3 "Memory"

	Unstandardised Coefficients		Standard. Coef.	t	Sig.
Variable	B	Std. Error	Beta		
(Constant)	5.766	.473		12.198	.000
Dist_Beachspot	.000	.000	-.145	-1.225	.223
Vis_Beachspot	-.085	.391	-.030	-.217	.828
Dist_Coastline	.002	.001	.587	2.564	.012
Vis_Coastline	.239	.229	.183	1.044	.299
Dist_CoastlineWN	4.664E-5	.000	.025	.175	.862
Vis_CoastlineWN	-.394	.332	-.244	-1.187	.238
Dist_Divespot	4.757E-5	.001	.013	.082	.935
Vis_Divespot	-.074	.301	-.046	-.245	.807
Dist_Corezone	.000	.000	-.213	-1.392	.167
Vis_Corezone	.324	.375	.125	.863	.390
Dist_Otherzone	-.002	.001	-.563	-2.393	.018
Vis_Otherzone	.102	.163	.088	.629	.531
Dist_Facilities	.000	.000	.175	1.332	.186
Vis_Facilities	.094	.122	.086	.765	.446
Dist_Road	-.001	.002	-.048	-.438	.662

Table S16. ANOVA test result from standard multiple regression analysis for model R3 "Memory"

ANOVA[a]

Model		Sum of Squares	df	Mean Square	F	Sig.
1	Regression	5.120	15	.341	1.150	.322[b]
	Residual	33.237	112	.297		
	Total	38.357	127			

a. Dependent Variable: MEM

b. Predictors: (Constant), Dist_Road, Vis_Beachspot, Dist_Beachspot, Dist_Divespot, Vis_Facilities, Vis_Divespot, Dist_CoastlineWN, Dist_Facilities, Vis_Otherzone, Dist_Corezone, Vis_Corezone, Dist_Coastline, Vis_Coastline, Vis_CoastlineWN, Dist_Otherzone

Table S17. T-test result from standard multiple regression analysis for model R4 "Place Identity"

	Unstandardised Coefficients		Standard. Coef.	t	Sig.
Variable	B	Std. Error	Beta		
(Constant)	5.728	.477		12.001	.000
Dist_Beachspot	.000	.000	-.085	-.712	.478
Vis_Beachspot	-.082	.395	-.029	-.207	.837
Dist_Coastline	.003	.001	.684	2.978	.004
Vis_Coastline	.265	.232	.202	1.146	.254
Dist_CoastlineWN	.000	.000	.216	1.536	.127
Vis_CoastlineWN	-.656	.335	-.404	-1.957	.053
Dist_Divespot	.000	.001	-.116	-.745	.458
Vis_Divespot	.376	.303	.231	1.238	.218
Dist_Corezone	.000	.000	-.226	-1.472	.144
Vis_Corezone	.044	.379	.017	.117	.907
Dist_Otherzone	-.003	.001	-.665	-2.817	.006
Vis_Otherzone	-.097	.164	-.083	-.590	.557
Dist_Facilities	.001	.000	.293	2.222	.028
Vis_Facilities	.270	.124	.245	2.185	.031
Dist_Road	.000	.002	-.021	-.189	.851

Table S18. ANOVA test result from standard multiple regression analysis for model R4 "Place Identity"

ANOVA[a]

Model		Sum of Squares	df	Mean Square	F	Sig.
1	Regression	4.953	15	.330	1.092	.372[b]
	Residual	33.884	112	.303		
	Total	38.838	127			

a. Dependent Variable: PLID

b. Predictors: (Constant), Dist_Road, Vis_Beachspot, Dist_Beachspot, Dist_Divespot, Vis_Facilities, Vis_Divespot, Dist_CoastlineWN, Dist_Facilities, Vis_Otherzone, Dist_Corezone, Vis_Corezone, Dist_Coastline, Vis_Coastline, Vis_CoastlineWN, Dist_Otherzone

Table S19. T-test result from standard multiple regression analysis for model R5 "Social"

	Unstandardised Coefficients		Standard. Coef.	t	Sig.
Variable	B	Std. Error	Beta		
(Constant)	5.975	.379		15.757	.000
Dist_Beachspot	.000	.000	-.188	-1.598	.113
Vis_Beachspot	-.227	.314	-.099	-.722	.472
Dist_Coastline	.002	.001	.683	3.012	.003
Vis_Coastline	.315	.184	.297	1.711	.090
Dist_CoastlineWN	.000	.000	.173	1.243	.216

Vis_CoastlineWN	-.387	.266	-.296	-1.454	.149
Dist_Divespot	.000	.000	-.049	-.316	.752
Vis_Divespot	.010	.241	.007	.040	.968
Dist_Corezone	.000	.000	-.313	-2.070	.041
Vis_Corezone	.277	.301	.132	.919	.360
Dist_Otherzone	-.002	.001	-.705	-3.026	.003
Vis_Otherzone	-.079	.131	-.084	-.607	.545
Dist_Facilities	.000	.000	.315	2.423	.017
Vis_Facilities	.168	.098	.190	1.712	.090
Dist_Road	-.001	.002	-.081	-.752	.453

Table S20. ANOVA test result from standard multiple regression analysis for model R5 "Social"

ANOVA[a]

Model		Sum of Squares	df	Mean Square	F	Sig.
1	Regression	3.792	15	.253	1.324	.200[b]
	Residual	21.390	112	.191		
	Total	25.182	127			

a. Dependent Variable: SOC

b. Predictors: (Constant), Dist_Road, Vis_Beachspot, Dist_Beachspot, Dist_Divespot, Vis_Facilities, Vis_Divespot, Dist_CoastlineWN, Dist_Facilities, Vis_Otherzone, Dist_Corezone, Vis_Corezone, Dist_Coastline, Vis_Coastline, Vis_CoastlineWN, Dist_Otherzone

Table S21. T-test result from standard multiple regression analysis for model R6 "Spiritual"

	Unstandardised Coefficients		Standard. Coef.	t	Sig.
Variable	B	Std. Error	Beta		
(Constant)	7.434	.497		14.959	.000
Dist_Beachspot	-.001	.000	-.313	-2.667	.009
Vis_Beachspot	-.257	.411	-.085	-.625	.533
Dist_Coastline	.002	.001	.531	2.346	.021
Vis_Coastline	.481	.241	.345	1.995	.048
Dist_CoastlineWN	.001	.000	.249	1.797	.075
Vis_CoastlineWN	-.225	.349	-.131	-.644	.521
Dist_Divespot	.000	.001	-.110	-.721	.473
Vis_Divespot	-.142	.316	-.083	-.451	.653
Dist_Corezone	-.001	.000	-.432	-2.865	.005
Vis_Corezone	.274	.395	.099	.693	.490
Dist_Otherzone	-.002	.001	-.509	-2.190	.031
Vis_Otherzone	-.193	.171	-.157	-1.129	.261
Dist_Facilities	.001	.000	.293	2.264	.026
Vis_Facilities	.130	.129	.112	1.010	.315
Dist_Road	-.003	.002	-.129	-1.194	.235

Table S22. ANOVA test result from standard multiple regression analysis for model R6 "Spiritual"

ANOVA[a]

Model		Sum of Squares	df	Mean Square	F	Sig.
1	Regression	6.735	15	.449	1.369	.175[b]
	Residual	36.740	112	.328		
	Total	43.475	127			

a. Dependent Variable: SPIR

b. Predictors: (Constant), Dist_Road, Vis_Beachspot, Dist_Beachspot, Dist_Divespot, Vis_Facilities, Vis_Divespot, Dist_CoastlineWN, Dist_Facilities, Vis_Otherzone, Dist_Corezone, Vis_Corezone, Dist_Coastline, Vis_Coastline, Vis_CoastlineWN, Dist_Otherzone

Table S23. T-test result from standard multiple regression analysis for model R7 "Therapeutic"

	Unstandardised Coefficients		Standard. Coef.	t	Sig.
Variable	B	Std. Error	Beta		
(Constant)	5.463	.416		13.139	.000
Dist_Beachspot	.000	.000	-.092	-.778	.438
Vis_Beachspot	-.163	.344	-.065	-.474	.636
Dist_Coastline	.001	.001	.450	1.980	.050
Vis_Coastline	.308	.202	.266	1.530	.129
Dist_CoastlineWN	.000	.000	-.065	-.470	.640
Vis_CoastlineWN	-.313	.292	-.219	-1.072	.286
Dist_Divespot	.000	.001	.119	.775	.440
Vis_Divespot	-.158	.264	-.110	-.596	.552
Dist_Corezone	.000	.000	-.165	-1.089	.278
Vis_Corezone	.261	.330	.114	.790	.431
Dist_Otherzone	-.002	.001	-.517	-2.218	.029
Vis_Otherzone	.023	.143	.022	.159	.874
Dist_Facilities	.000	.000	.195	1.502	.136
Vis_Facilities	.084	.108	.087	.782	.436
Dist_Road	-.001	.002	-.069	-.640	.523

Table S24. ANOVA test result from standard multiple regression analysis for model R7 "Therapeutic"

ANOVA[a]

Model		Sum of Squares	df	Mean Square	F	Sig.
1	Regression	4.473	15	.298	1.299	.215[b]
	Residual	25.714	112	.230		
	Total	30.187	127			

a. Dependent Variable: THEU

b. Predictors: (Constant), Dist_Road, Vis_Beachspot, Dist_Beachspot, Dist_Divespot, Vis_Facilities, Vis_Divespot, Dist_CoastlineWN, Dist_Facilities, Vis_Otherzone, Dist_Corezone, Vis_Corezone, Dist_Coastline, Vis_Coastline, Vis_CoastlineWN, Dist_Otherzone

Table S25. Communalities values for Model E1.

Communalities		
	Initial	Extraction
Eng1	1.000	.559
Eng2	1.000	.575
Eng4	1.000	.647
Plid6	1.000	.544
Plid7	1.000	.534
Theu9	1.000	.684
Theu10	1.000	.694
Theu11	1.000	.597
Spir12	1.000	.446
Spir13	1.000	.782
Spir14	1.000	.852
Soc15	1.000	.726
Soc16	1.000	.535
Mem18	1.000	.677
Mem19	1.000	.570
Mem20	1.000	.624
Skill21	1.000	.624
Skill22	1.000	.604
Skill23	1.000	.495
Extraction Method: Principal Component Analysis.		

Table S26. The goodness of fit indicators used for Model E1.

	SRMR	CMIN (χ2)	df	CMIN/df	CFI	TLI	RMSEA
**Threshold**	< 0.08	≠ 0	≠ 0	1<x<3	> 0.90	> 0.90	< 0.08
**Model**	0.073	282.334	131	2.155	0.872	0.833	0.094

Table S27. Factor score weight produced for Model E1 Gili Matra.

	SKILL	*MEM*	*SOC*	*SPIR*	*THEU*	*PLID*	*ENG*
Eng4	0.02	-0.005	0.015	0.004	0.01	0.04	0.076
Skill21	0.153	0.069	0.035	-0.007	0.032	0.001	0.041
Skill22	0.104	0.047	0.024	-0.004	0.022	0.001	0.028
Skill23	0.078	0.035	0.018	-0.003	0.017	0.001	0.021
Mem18	0.047	0.223	0.038	0.011	0.021	0.021	-0.007
Mem19	0.035	0.167	0.028	0.008	0.016	0.016	-0.006
Mem20	0.021	0.099	0.017	0.005	0.009	0.009	-0.003
Soc15	0.032	0.05	0.012	0.059	0.044	0.211	0.027
Soc16	0.012	0.019	0.004	0.022	0.016	0.078	0.01
Spir12	-0.003	0.006	0.025	0.073	0.003	-0.006	0.003
Spir13	-0.007	0.016	0.065	0.187	0.008	-0.015	0.009

Spir14	-0.022	0.055	0.221	0.641	0.028	-0.05	0.03
Theu9	0.029	0.028	0.043	0.007	0.222	-0.012	0.019
Theu10	0.036	0.034	0.054	0.009	0.275	-0.015	0.023
Theu11	0.011	0.011	0.017	0.003	0.085	-0.005	0.007
Plid6	0.001	0.016	0.121	-0.008	-0.007	0.261	0.042
Plid7	0	0.012	0.09	-0.006	-0.005	0.194	0.031
Eng1	0.032	-0.008	0.023	0.007	0.016	0.063	0.12
Eng2	0.041	-0.011	0.03	0.009	0.021	0.081	0.154
